# High Spending on Maternity Care in India: What Are the Factors Explaining It?

**DOI:** 10.1371/journal.pone.0156437

**Published:** 2016-06-24

**Authors:** Srinivas Goli, Moradhvaj, Anu Rammohan, Shruti, Jalandhar Pradhan

**Affiliations:** 1 Centre for the Study of Regional Development (CSRD), School of Social Sciences (SSS), Jawaharlal Nehru University (JNU), New Delhi, India; 2 Discipline of Economics, University of Western Australia, Crawley, WA, Australia; 3 Discipline of Economics, Faculty of Social Sciences, Banaras Hindu University (BHU), Varanasi, Uttar Pradesh, India; 4 Department of Humanities & Social Sciences, National Institute of Technology, Rourkela, Orissa, India; University of Missouri, UNITED STATES

## Abstract

**Background and Objectives:**

High maternity-related health care spending is often cited as an important barrier in utilizing quality health care during pregnancy and childbirth. This study has two objectives: (i) to measure the levels of expenditure on total maternity care in disaggregated components such as ANCs, PNCs, and Natal care expenditure; (ii) to quantify the extent of catastrophic maternity expenditure (CME) incurred by households and identify the factors responsible for it.

**Methods and Findings:**

Data from the 71^st^ round of the National Sample Survey (2014) was used to estimate maternity expenditure and its predictors. CME was measured as a share of consumption expenditure by different *cut-offs*. The two-part model was used to identify the factors associated with maternity spending and CME. The findings show that household spending on maternity care (US$ 149 in constant price) is much higher than previous estimates (US$ 50 in constant price). A significant proportion of households in India (51%) are incurring CME. Along with economic and educational status, type of health care and place of residence emerged as significant factors in explaining CME.

**Conclusion:**

Findings from this study assume importance in the context of an emerging demand for higher maternity entitlements and government spending on public health care in India. To reduce CME, India needs to improve the availability and accessibility of better-quality public health services and increase maternity entitlements in line with maternity expenditure identified in this study.

## Introduction

Over the last two decades, efforts to reduce maternal mortality have remained at the policy forefront in many developing countries and continue to occupy considerable attention as evidenced by Goal-3 of the recently proposed Sustainable Development Goals (SDGs) [1, 2]. Despite a 45% decline in global maternal mortality burden since 1990, maternal mortality ratios in developing countries remain a significant challenge to health systems. Nearly 99% of all maternal deaths occur in developing countries [3]. Although India has achieved significant progress in the reduction of Maternal Mortality Rates (MMR) over the last decade, from 560 per 100,000 live births in 1990 to 167 per 100,000 live births in 2011–13, it remains the highest contributor in terms of absolute numbers of female deaths occurring in the world due to pregnancy-related causes. In 2014, India along with Nigeria accounted for one-third of all global maternal deaths, with 17% (50,000) in India and 14% (40,000) in Nigeria [3, 4, 5].

Further, despite considerable economic progress, public expenditure on healthcare in India has stagnated since the early 1990s. Government expenditure on health care is considerably low compared to other countries with similar levels of per-capita income, inequality and poverty [6, 7]. On the other hand, the private healthcare industry has witnessed an unprecedented boom, growing at a rate of over 15% compounded annually, more than twice the growth rate for all services over the same period [8]. As a consequence, the pattern of current health spending suggests that households meet 71% of their total health spending, with Government contributions accounting for only 20% (Central), 6% (State) and the remaining 2% made up by local bodies, other firms and external flows respectively [9].

In order to reduce Out-Of-Pocket Expenditures (OOPE) on maternal health care services, and improve maternal health outcomes in the country, the Government of India and some of the state governments have implemented demand-side financing schemes (e.g. *Janani Suraksha Yojana (JSY)*, *Agra Voucher Scheme*, *MAMATA scheme*) [9, 10]. In particular, the *JSY* the world’s largest Conditional Cash Transfer (CCT) scheme under the National Health Mission (NHM), is being implemented with the aim of reducing financial barriers in maternal health care services. However, the grossly insufficient provision of quality health care services by the Government, and the high cost of private health care for quality maternity care services [Antenatal services (ANCs), Natal and Postnatal services (PNCs)] is hampering safe motherhood initiatives in India [10, 11, 12, 13, 14, 15]. Also, government health services in India are marred by unavailability and absenteeism of health professionals, poor health care facility and infrastructure, shortage of drugs and equipment, physical inaccessibility and some anecdotal evidence of callous behaviour of healthcare professionals. These factors have contributed to an increase in the proportion of women attracted towards private maternal health care services [16, 17, 18, 19]. Previous studies have pointed to an increase in the proportion of cesarean section births that contribute to higher maternity expenditures [20, 5].

High OOPE are pushing a considerable proportion of households below the poverty line, a well-acknowledged fact in the health economics literature [21, 22, 23, 8]. However, it is only in the recent years that researchers have started exploring the question: could maternal healthcare related expenditures become catastrophic for households? Along with other developing countries, researchers in India have also attempted to answer this question. Many of these studies suggest that the cost of ANCs and delivery (obstetric) care could be catastrophic in low-income settings [24, 14, 25, 26].

However, the studies on maternity spending in India have serious limitations. For example, studies conducted by Skordi-Worral et al. [14] and Sambo et al. [26] were based on local level evidence with small samples and their findings cannot be generalised easily to the national level. Others such as Bonu et al. [24] and Mohanty and Srivastava [25] have used representative samples nationally, but their data suffers from serious limitations, which can have implications for their findings. For instance, Bonu et al. [24] have assessed the 60^th^ round of the National Sample Survey (NSS) to estimate prenatal, postnatal and childbirth expenditures. In the 60^th^ round of the NSS, information on maternal health care costs were collected at an aggregate-level based on a simple question for each of the ANCs, PNCs, and Natal care cost. Therefore, there is a greater chance of under-reporting of expenditures [27]. On the other hand, studies by Lim et al. (2010) and Mohanty and Srivastava [25] conducted their analysis using the nationally representative District Level Household Survey (DLHS). However, a key shortcoming of these studies is that they restricted their analyses to only delivery care expenditures, because DLHS does not have any information related to expenditures on ANCs and PNCs. Another important limitation of the DLHS data is that the information on delivery expenditures was collected for the last birth in the five years preceding the survey. As pointed out previously, this information is subject to potential recall bias [27].

Therefore, the aim of this paper is to: (i) quantify the Total Maternity Expenditure (TME) incurred by households by disaggregated components such as ANCs, PNCs, and Natal care, and to analyse its association with the socio-economic characteristics of the households; (ii) assess the proportion of households incurring Catastrophic Maternity Expenditure (*hereafter* CME) by different *cut-offs*. The different definitions used for estimating CME and their purpose are explained in detail in the following section.

## Methods

### Data

The data used in this study comes from the 25^th^ schedule of the 71^st^ round of the Indian National Sample Survey Organisation—titled “*Key Indicators of the Social Consumption in India*: *Health*”, conducted between January to June 2014. The survey selected a nationally representative sample of 65,932 households with 333,104 persons across various States and Union Territories in India. Of these, 36,480 households were from 4,577 rural villages, and 29,542 households were from 3,720 urban blocks. The NSS uses a stratified two-stage design in the sampling of census villages in the rural areas and the NSS urban frame survey blocks in the urban areas in the first stage, followed by a sampling of households in the second stage [28].

Data on ANCs and PNCs expenditures were collected from women aged 15–49 years who delivered the baby or were pregnant in the 365 days prior to the survey. Information on delivery care expenditure was collected as expenses incurred during the last 365 days for in-patient medical care during childbirth. In all, 19,445 women reported being pregnant in the 365 days prior to the survey, of whom 14,482 women reported of having a hospital birth during the reference period. Since the aim of this study is to analyse pregnancy and maternity expenditure, we restrict our analysis to those 14,482 women who gave birth in a hospital setting. Within this sample, only 13,596 women have utilised any ANCs, while only 10,798 women have had PNCs. Therefore, for analyses related to total maternity spending, we have included only those women who accessed all three maternity care services: those who delivered in a hospital and who have also accessed both ANCs and PNCs services (n = 10,379).

Unlike the earlier rounds of NSS, in the 71^st^ round, information on delivery care expenditure was collected at a disaggregated level in eight sub-components: package components, doctor’s or surgeon’s fee (hospital staff or other specialists), medicines, diagnostic tests, bed charges, other medical expenses (attendant charges, charges related to physiotherapy, personal medical appliances, blood, oxygen), transport cost for patient, other non-medical expenses incurred by the household on food, transport cost for others, expenditure on escort, and lodging charges if any. However, information on ANCs and PNCs was collected as aggregate expenditures. Maternity expenditure in all three components were collected by the source of health care (public or private) for ANCs, PNCs, and delivery care. For the collection of both household consumption expenditures and maternal expenditures, efforts were made to interview all the adult male members personally to take care of potential underestimation if only women were interviewed [28].

Information on household consumption expenditure was collected through a short set of five questions on consumption aggregates, rather than a detailed listing of consumption items, in the 30 days prior to the survey. The five items used for calculating the household consumption expenditure were: (i) purchases; (ii) home-produced stock; (iii) receipts from the exchange of goods and services; (iv) gifts and loans; and (v) free collection. This rather crude determination of total household expenditure from relatively few consumption aggregates over a 30-days reference period remains a limitation of this study. The survey also collected detailed information on the socio-economic and demographic characteristics of the households, the nature and the level of utilization of health care for household members suffering from any ailments.

### Ethics Statement

The unit level data from the NSS 71^st^ round on social consumption relating to health is widely accepted and is considered to be reliable. It was conducted by the office of the National Sample Survey Organization under the aegis of Ministry of Statistics and Program Implementation, Government of India. Ethical approval for the survey was obtained at two levels: First, the ethical approval for the survey was obtained from the National Sample Survey Office. Second, a standard consent form approved by the ethics review committee was read out to the respondent in their native language. Once the respondent agreed to participate in the survey, the interviewer got the consent form signed form respondent acknowledging that he/she had read the form, had understood the purpose of the study and agreed to participate. The information collected in the survey is used primarily for research and the name and place of the respondents have not been disclosed. The dataset used in this study is also available in the public domain [28].

### Measure

#### Outcome variables

The main dependent variables in this analysis include four measures relating to different aspects of maternity expenditure: ANCs, PNCs, delivery and TME. Additionally, we also examine whether the household incurred any CME. Expenditure relating to ANCs, delivery and PNCs includes expenses incurred in obtaining these services, whereas the TME includes the expenditures on all three components (ANCs, delivery, and PNCs). To avoid complexity in the multivariate models, we have not carried out detailed analyses of expenditure by its disaggregated components. We used the standard method of estimating catastrophic expenditure in measuring CME. There are two widely used methods in the health economics literature in measuring catastrophic expenditure [29, 30, 31, 32, 23, 33, 34, 24]. The first method categorizes the proportion of households with catastrophic health expenditure based on the share of health expenditure in the household’s total consumption expenditure with different *cut-offs*. The second method compares the health expenditure to the household’s ‘*capacity to pay’*, calculated as total annual expenditure minus subsistence expenditure on food and other basic needs. Due to the absence of disaggregated data on individual expenditure items such as food and non-food in this survey, the calculation of ‘*capacity to pay’* necessitates a different approach [23, 33]. However, given the context of unsettled debates and disagreements in defining the basic consumption requirements and poverty line in India [35, 36], we have chosen not to use the second method to calculate the catastrophic health expenditure. Accordingly, we define CME using the first method but with more than one *cut-off* (5%, 10%, and 15%). The three *cut-offs* used in this study indicate the catastrophic spending at low (5%), medium (10%) and high (15%) variants. Also, the use of different *cut-offs* gives us an opportunity to assess the severity or intensity of the problem [33].

#### Explanatory variables

The explanatory variables were selected based on existing literature as well as social, cultural and political aspects specific to India [29, 33, 24, 37, 38, 26]. The variables that are important markers of social and economic disparities in India include female education level, social groups (as measured by *Caste)*, religion and economic/income status (as measured by household’s Monthly Per Capita Consumption Expenditure [MPCE]). In particular, *Caste* is viewed as a distinctive sociological imagination of unadulterated status by birth, based on religion and ideological grounds. The traditional Hindu Varnas (translated into English as *Castes*) were five, Brahmins (priests, teachers), Kshatriyas (warriors, royalty), Vaisyas (money lender, traders), and the Sudras (menial job) and the Ati Sudras and *Dalits* (the untouchables, doing lowest of the menial jobs). There are thousands of sub-castes within these five *Castes*. However, for the political and developmental planning and policy perspective, the constitution of India classified traditional *Caste* groups into four broad categories: scheduled castes (SCs), scheduled tribes (STs), other backward castes (OBCs) and general castes) [6, 38, 39]. We have also used variables relating to demographic and maternal health care characteristics of women such as age, information about the previous and the current pregnancy, health insurance coverage and whether the care provider is public or private.

#### Statistical Methods

Descriptive statistics were used to describe the nature of the variables. The bivariate analyses examined the unadjusted association of various independent variables on the outcome variables as defined earlier. A two-part multivariate regression model was fitted to assess the independent association of the outcome variables with the explanatory variables. The two-part model is the most straightforward approach to use in this context, and is widely used when the outcome variable (health expenditure) takes on a value of zero for a substantial number of cases [40]. In its most popular form, this comprises a Probit model to determine the probability that an individual makes any catastrophic expenditure and an OLS model to determine non-zero expenditures on maternity care. Here, we assume that there is an unobserved variable y^*^ that is generated from the following model:

$$y^{*}=\beta x+e$$ (1)

Where *ß* is a K-vector of parameters, *x* is a vector of explanatory variables, and *e* ∼ *N*(0,1) is a random shock. We observe *y* = 1 if y^*^>0 and *y* = 0 otherwise.

However, to interpret the quantitative implication of the results, we computed the average marginal effects of binary explanatory variables. Unlike the linear probability model, the average marginal effects are not given by the coefficients directly, but they were computed from the coefficients. The formula for the average marginal effect of an explanatory variable x_**k**_ is given by:

$$\text{P}(\text{y}=1|{\text{x}}_{k}=1)-\text{P}(\text{y}=1|{\text{x}}_{k}=0)=\text{F}(\text{x}\beta |{\text{x}}_{k}=1)-\text{F}({\text{x}\beta |\text{x}}_{k}=0)$$ (2)

Where y = 1 if there is catastrophic maternity expenditure, otherwise 0. Average marginal effects depend on the values of the x_k_ variables, where k represents education, MPCE quintile, type of hospital and other factors in the model.

As previously mentioned, in the second part an OLS model was applied only to the sub-sample with non-zero expenditures, in order to estimate the correlates of the positive levels of expenditure. The OLS model is often considered to be adequate for analyses of health sector inequalities, where we simply want to predict, for example, maternity care expenditure conditional on income, age, and social affiliation so on. Given that, typically the distribution of maternity care expenditures is right-skewed, invariably the log of expenditure is modeled as a part of OLS.

Following Jones (2000), we assume that the probability of incurring maternity expenditure (*y*_*i*_ > 0) is positive and determined by observable (*X*_1*i*_) and unobservable (ε_1*i*_) factors. This can be represented in an equation as below.

$$E[\text{ln}(y_{i})|y_{i}>0,X_{2i}\beta_{2}]=E[\text{ln}(y_{i})|X_{1i}\beta_{1}+\varepsilon_{1i}>0,X_{2i}\beta_{2}]=X_{2i}\beta_{2}$$ (3)

Where *ln* (*y*_*i*_ > 0) is the log of positive maternity expenditure, *X*_2*i*_, refers to a vector of covariates the term ε_2*i*_ includes unobservable factors, *E* is the expected level of medical expenditure.

## Results

### Descriptive Statistics of the Study Variables

The average *Cost* of ANCs and PNCs per pregnancy were US$ 46 and US$ 28 respectively, with delivery expenditure (US$ 160) accounting for the largest share of average TME (US$ 258). The catastrophic maternity spending of the households depend on the *cut-off* chosen for the CME definition. For example, at the 5% threshold level, 75% of the households incurred CME. The proportion reduced to 50% at the 10% threshold level and further decreased to 34% at 15% level [Table 1].

**Table 1:** Descriptive statistics of the study variables (n = 14482).

Variable	Categories	Proportion/ mean	±95% CI	Standard Deviation	Minimum	Maximum	Mean
Outcome Variables		Rupees					US$
**Total ANC expenditure (n = 13596)**		2790.7	±90.85	7947.4	0	675000	45.94
**Total PNC expenditure (n = 10798)**		1714.2	±54.75	3470.2	0	85000	28.22
**Delivery expenditure (n = 14482)**		9701	±231.00	14519	0	347000	159.69
***Medical expenditure***		8155.3	±216.15	13795.5	0	336500	134.24
***Transport expenditure***		584.1	±11.65	694.8	0	21000	9.61
***Other non-medical expenditure***		1040.5	±22.35	1262.3	0	26760	17.13
**Total spending on Pregnancy and Child Birth (n = 10379)**		15658.4	±344.60	17910.9	0	695900	257.75
**Households with CME (n = 10379)**	At 5% *cut-off*	0.7591	±0.0155				
	At 10% *cut-off*	0.5139	±0.0190				
	At 15% *cut-off*	0.3431	±0.0175				
**Predictors**							
**Age (in years)**	15–24	0.4019	±0.0080				
	25–29	0.3512	±0.0078				
	30–49	0.2469	±0.0070				
**Place of residence**	Rural	0.5549	±0.0081				
	Urban	0.4451	±0.0081				
**Education Level of women**	No education	0.2278	±0.0068				
	Primary	0.2316	±0.0069				
	Secondary	0.1718	±0.0061				
	Higher secondary	0.2475	±0.0070				
	Graduate and above	0.1213	±0.0053				
**Religion**	Hindu	0.7741	±0.0068				
	Muslim	0.1396	±0.0056				
	Others	0.0863	±0.0046				
**Social group**	SC/ST	0.2988	±0.0075				
	OBC	0.4027	±0.0080				
	General caste	0.2985	±0.0075				
**MPCE quintile**	Poorest	0.2471	±0.0070				
	Poorer	0.1566	±0.0059				
	Middle	0.2108	±0.0066				
	Richer	0.1901	±0.0064				
	Richest	0.1954	±0.0065				
**Serial number of the pregnancy**	One	0.9979	±0.0008				
	Two or more	0.0021	±0.0008				
**Type of Health Facility**	Public	0.5336	±0.0084				
	Private	0.3322	±0.0079				
**Insurance**	No	0.8954	±0.0101				
	Yes	0.1046	±0.0101				
**Region**	North	0.1458	±0.0058				
	Central	0.1587	±0.0060				
	East	0.1805	±0.0063				
	Northeast	0.1271	±0.0054				
	West	0.1364	±0.0056				
	South	0.1983	±0.0065				
	Union Territories	0.0533	±0.0037				

Furthermore, sample distribution by socioeconomic and demographic characteristics shows that about 40% of the sample were below 25 years while 35% were in the age group of 25–29 years, and 25% in the age group of 30–49 years. The majority of the sample lives in rural areas. It was also observed that nearly 23% of the sample were illiterate while 23% had completed primary schooling, 17% with secondary schooling, 25% up to higher secondary and 12% have studied up to graduation and above. In terms of religion, 77% of the women sample belonged to the Hindu community, 14% to Muslims and 8% to other religious groups, includes Christians, Sikhs, Jains and Buddhist, etc. By social group (*Caste* affiliation) of women, the results show that in the full sample, 40% belonged to OBC), 30% belonged to SCs/ STs, and 30% belong to General *Caste*. The distribution of the sample in terms of the type of health care use shows that respondents were predominantly using public health facilities for pregnancy and maternity care. With regards to health insurance coverage, only 10% of the sample was covered by any formal insurance scheme.

### OOPE for Maternity Care by Background Characteristics

The average OOPE incurred by different socio-economic and demographic characteristics of the women shows that an increase in age is associated with incurring higher mean expenses on ANCs and PNCs, while spending on delivery expenditure and total maternity expenditure are non-linear, increasing and then decreasing (Table 2 and S1 Table). Average spending on total maternity cost was US$235 among women in the age group of 15–24 years, US$ 241 in the age-group 25–29 years, falling to US$ 225 for women aged 30–49 years. Urban women incurred higher maternity expenditure (ANCs = US$ 67, delivery care = US$ 215, PNCs = US$35, total maternity care = US$ 326) than their rural counterparts (ANC = US$ 37; delivery = US$ 139; PNC = US$ 25; total maternity care = US$ 196). Similarly, women who were educated, in particular those with education levels up to graduation and above (ANC = US$ 58, PNC = US$ 32, delivery care = US$ 322, total maternity care = US$ 445) were spending more while the lowest spending was for illiterate women (ANC = US$ 38, PNC = US$ 28, delivery care = US$ 101, total maternity care = US$ 168). Hindu women were spending less than Muslims and others (Table 2 and S1 Table). By *Caste* affiliation of the women, the results show that absolute spending on maternity care among the women from the General *Caste* is higher than their two other socially disadvantaged groups (SC/ST and OBC). Based on economic status of women, the mean expenditure on maternity care increases with an increase in the household’s economic status: with women from the richest income group spending significantly higher (ANCs = US$ 55, PNCs = US$ 30, delivery care = US$ 290, total maternity care = US$ 365) than the other economic groups. Women were spending less on maternity care for their second or higher order pregnancies than their first pregnancy. Spending on maternity care at private health centers (ANCs = US$ 85, PNCs = US$ 44, delivery care = US$ 174, total maternity care = US$ 305) was in general higher than in public health care facilities (ANC = US$ 35, PNC = US$ 20, delivery care = US$ 144, total maternity care = US$ 197).

**Table 2:** Mean spending (US$) on maternity care services.

Variable	Categories	Prenatal (n = 13596)		Postnatal care (n = 10798)		Delivery cost (n = 14482)		Total maternity cost (n = 10379)	
		Mean	±95% CI	Mean	±95% CI	Mean	±95% CI	Mean	±95% CI
**Age (in years)**	15–24	43.71	±4.05	26.89	±2.06	161.97	±13.77	235.43	±16.80
	25–29	47.65	±3.18	29.88	±2.00	164.64	±19.33	241.59	±11.57
	30–49	48.03	±5.51	28.29	±2.48	147.80	±12.11	224.91	±14.45
**Place of residence**	Rural	37.34	±2.98	25.54	±1.54	138.57	±11.84	196.28	±11.18
	Urban	67.43	±3.81	34.66	±2.17	215.12	±13.08	325.95	±12.20
**Education Level of women**	No education	37.97	±3.81	27.86	±3.00	101.27	±10.32	167.75	±11.63
	Primary	47.41	±5.80	27.27	±2.09	139.05	±13.36	212.15	±14.47
	Secondary	42.79	±4.85	26.90	±3.00	148.05	±30.25	204.13	±14.65
	Higher secondary	50.19	±5.50	28.37	±2.30	181.62	±20.64	254.83	±13.34
	Graduate and above	57.69	±7.28	32.56	±4.25	322.25	±39.65	445.27	±54.91
**Religion**	Hindu	44.65	±2.69	27.72	±1.46	159.85	±10.08	234.88	±10.72
	Muslim	50.16	±7.44	28.94	±2.95	159.53	±34.99	219.88	±14.82
	Others	53.13	±7.54	32.26	±3.95	158.22	±14.92	265.29	±22.27
**Social group**	SC/ST	40.92	±3.50	29.97	±2.78	108.60	±19.58	175.78	±20.71
	OBC	45.65	±3.57	27.29	±1.82	178.12	±14.76	245.60	±11.73
	General caste	51.73	±5.49	27.73	±1.94	189.48	±13.85	282.77	±14.29
**MPCE quintile**	Poorest	41.29	±4.11	27.77	±3.10	78.90	±7.26	153.43	±10.14
	Poorer	43.27	±6.23	26.92	±2.83	137.63	±32.10	210.23	±36.91
	Middle	47.71	±6.38	27.70	±2.48	143.37	±11.70	215.89	±13.74
	Richer	44.46	±5.38	28.82	±2.79	187.95	±17.51	261.59	±15.79
	Richest	55.03	±4.81	30.09	±2.52	290.63	±30.11	365.28	±19.20
**Serial number of the pregnancy**	One	45.96	±2.43	28.18	±1.26	159.67	±9.30	235.20	±8.94
	Two or more	28.14	±15.06	95.81	±120.19	171.96	±120.88	111.77	±36.01
**Type of Health Facility**	Public	35.03	±3.01	20.02	±1.21	143.67	±7.01	197.30	±8.13
	Private	84.93	±5.83	44.08	±2.94	174.26	±10.13	305.98	±13.27
**Insurance**	No	45.71	±5.87	27.24	±2.35	143.13	±14.32	219.52	±12.60
	Yes	56.72	±11.25	36.62	±10.25	323.79	±60.93	439.83	±63.81
**Region**	North	43.88	±4.56	22.59	±2.73	116.90	±11.29	199.13	±14.18
	Central	49.18	±7.66	26.87	±3.24	108.84	±16.28	174.42	±16.24
	East	48.97	±7.23	28.76	±4.22	124.88	±26.55	216.92	±41.83
	Northeast	57.36	±7.45	38.89	±5.43	120.21	±11.37	234.60	±18.31
	West	41.87	±6.09	31.11	±3.29	189.79	±22.83	246.43	±19.06
	South	46.22	±5.04	27.79	±2.17	215.30	±24.53	284.93	±16.27
	Union Territories	35.00	±7.11	28.16	±5.10	190.62	±26.00	251.18	±33.21
**Total**		45.94	±2.43	28.22	±1.26	159.69	±9.29	235.17	±8.94

### Household Catastrophic Expenditure

The results from catastrophic spending at different thresholds estimated by independent variables are presented in Table 3. The results reveal that although women in the age group 30–49 years spent less than other age groups, the difference was not large. However, the rural-urban difference in the proportion of households with catastrophic spending was high across all three definitions used. For instance, at the 10% *cut-off*, the proportion of households that incurred catastrophic spending was significantly higher in urban areas (58%) than in rural areas (48%). With the same *cut-off*, women in households from other than Hindu and Muslim religious community and those who belonged to General *Caste* were having higher catastrophic expenditures on maternity care than their counterparts. Marked differences were observed in catastrophic spending on maternity care between the poorest and the richest MPCE quintile households by all three definitions used. At the 10% *cut-off*, households in the poorest MPCE quintile faced 27% higher probability of incurring catastrophic spending on maternity care than households in the richest MPCE. With the same definition, similar differences were also observed by type of health facility used for delivery. It was also observed that the proportion of households with catastrophic expenditure was significantly higher in private health care facilities (62%) than government health care facilities (46%).

**Table 3:** Percentage share of total maternity expenditure in annual household consumption expenditure by different *cut-offs* and background characteristics (n = 10379).

Variable	Categories	At 5% *cut-off*		At 10% *cut-off*		At 15% *cut-off*	
		Proportion	± 95% CI	Proportion	± 95% CI	Proportion	± 95% CI
**Age (in years)**	15–24	0.7550	±0.0242	0.5030	±0.0310	0.3373	±0.0072
	25–29	0.7781	±0.0244	0.5536	±0.0298	0.3718	±0.0076
	30–49	0.7382	±0.0346	0.4747	±0.0373	0.3108	±0.0079
**Place of residence**	Rural	0.7333	±0.0210	0.4822	±0.0254	0.3138	±0.0057
	Urban	0.8192	±0.0170	0.5879	±0.0225	0.4115	±0.0061
**Education Level of women**	No education	0.7211	±0.0336	0.4305	±0.0364	0.2848	±0.0083
	Primary	0.7525	±0.0336	0.4924	±0.0360	0.3348	±0.0084
	Secondary	0.7421	±0.0374	0.4993	±0.0453	0.3314	±0.0118
	Higher secondary	0.7875	±0.0297	0.5796	±0.0406	0.3589	±0.0095
	Graduate and above	0.8209	±0.0330	0.6244	±0.0465	0.4808	±0.0131
**Religion**	Hindu	0.7578	±0.0175	0.5101	±0.0207	0.3454	±0.0050
	Muslim	0.7617	±0.0474	0.5218	±0.0662	0.3154	±0.0129
	Others	0.7686	±0.0418	0.5414	±0.0502	0.3658	±0.0121
**Social group**	SC/ST	0.7197	±0.0303	0.4511	±0.0333	0.2862	±0.0079
	OBC	0.7682	±0.0231	0.5315	±0.0279	0.3683	±0.0069
	General caste	0.7872	±0.0280	0.5543	±0.0378	0.3656	±0.0088
**MPCE quintile**	Poorest	0.8545	±0.0246	0.6483	±0.0354	0.4744	±0.0096
	Poorer	0.8168	±0.0398	0.5439	±0.0503	0.3728	±0.0124
	Middle	0.7404	±0.0375	0.5004	±0.0489	0.3095	±0.0100
	Richer	0.7135	±0.0335	0.4560	±0.0369	0.2861	±0.0080
	Richest	0.6455	±0.0363	0.3819	±0.0335	0.2390	±0.0073
**Serial number of the pregnancy**	One	0.7591	±0.0155	0.5140	±0.0190	0.3431	±0.0045
	Two or more	0.7128	±0.4138	0.1555	±0.2686	0.0422	±0.0239
**Type of Health Facility**	Public	0.7055	±0.0223	0.4605	±0.0268	0.2837	±0.0055
	Private	0.8700	±0.0185	0.6235	±0.0264	0.4470	±0.0070
**Insurance**	No	0.7749	±0.0353	0.5501	±0.0470	0.3430	±0.0103
	Yes	0.8608	±0.0633	0.6076	±0.0861	0.4439	±0.0231
**Region**	North	0.6517	±0.0367	0.3887	±0.0413	0.2358	±0.0412
	Central	0.6534	±0.0481	0.4068	±0.0457	0.2732	±0.0397
	East	0.8458	±0.0315	0.5876	±0.0568	0.3508	±0.0521
	Northeast	0.8522	±0.0293	0.6350	±0.0406	0.4363	±0.0432
	West	0.7588	±0.0431	0.4878	±0.0499	0.3064	±0.0418
	South	0.8099	±0.0290	0.5856	±0.0365	0.4220	±0.0367
	Union Territories	0.7619	±0.0683	0.5449	±0.0740	0.4193	±0.0722

### Adjusted Effects of Independent Factors

Table 4 shows the adjusted effects of independent factors on the probability of household’s participation in catastrophic spending and positive spending on maternity expenditure using a two-part model. The Predicted Probability (PP) of having CME at the 5%, 10% and 15% *cut-offs* reveal that irrespective of the *cut-offs*, the variation in catastrophic spending is sustained across women from different socioeconomic background characteristics. The probability of participation in CME at the 10% *cut-off* (mid-variant) by background characteristics, shows that variables such as place of residence, education of women, social group, economic status, type of health facility and region are statistically significant predictors of spending on maternity care in India. However, the maternity differences were more pronounced by the respondent’s education and economic categories. An increase in education from being in the no education category to graduation and above, increases the probability of having CME by five percentage points after controlling for key factors like economic status, type of health care and place of residence. It is possible that despite having the same economic status, women with higher education show a greater willingness to pay for health care than lower educated women. However, after controlling for other factors, an increase in economic status from lowest to highest quintile decreases the probability of having catastrophic spending by 47 percentage points after controlling for other factors. Similarly, delivery in a private hospital increases the probability of catastrophic spending by 46 percentage points compared to delivery in the public hospital.

**Table 4:** Two part model estimates showing the factors affecting the total maternity expenditure in different *cut-offs (*n = 10379).

		Total Maternity Expenditure				
Variable	Categories	Participation in Catastrophic Payments			Continuous spending on Maternity	
		(Predicted Probabilities from Probit model)			(Predicted Means of spending on Maternity care from OLS Model)	
		At 5% *cut-off*	At 10% *cut-off*	At 15% *cut-off*	US $	Rs.
**Age (in years)**	15–24	0.7682±0.0112	0.5198±0.0131	0.3527±0.0126	225.84±3.83	13720±233
	25–29	0.7848**±0.0117	0.5495***±0.0138	0.3757**±0.0135	247.31***±4.12	15024***±250
	30–49	0.7792±0.0139	0.5493***±0.0165	0.3766**±0.0163	246.27***±4.91	14961***±298
**Place of residence**	Rural	0.7401±0.0103	0.4867±0.0119	0.3248±0.0112	201.17±2.87	12221±174
	Urban	0.8183***±0.0101	0.5939***±0.0125	0.4130***±0.0126	332.95***±3.77	20227***±229
**Education Level of women**	No education	0.7587±0.0153	0.4978±0.0186	0.3317±0.0179	177.22±4.18	10766±254
	Primary	0.7794**±0.0143	0.5318***±0.0173	0.3624**±0.0168	220.46±4.46	13393±271
	Secondary	0.7734±0.0168	0.5265**±0.0201	0.3700***±0.0198	210.04±5.15	12760±313
	Higher secondary	0.7951***±0.014	0.5694***±0.0168	0.3807***±0.0164	257.4**±4.64	15637**±282
	Graduate and above	0.7772±0.0227	0.5747***±0.0248	0.4024***±0.0243	427.82***±6.54	25990***±397
**Religion**	Hindu	0.7786±0.0079	0.5409±0.0094	0.3691±0.0092	238.73±2.78	14503±169
	Muslim	0.7841±0.0193	0.533±0.0225	0.3601±0.0219	221.02***±6.4	13427***±389
	Others	0.7471**±0.0272	0.5196±0.0305	0.3606±0.0294	252.72±8.39	15353±510
**Social group**	SC/ST	0.7592±0.0137	0.5175±0.0165	0.3544±0.0162	179.93±3.72	10931±226
	OBC	0.7703±0.0114	0.528±0.0134	0.3625***±0.0130	249.83±3.81	15177±232
	General caste	0.8051***±0.013	0.5707***±0.0156	0.3847**±0.0154	283***±4.69	17192***±285
**MPCE quintile**	Poorest	0.8996±0.0099	0.7565±0.0139	0.6174±0.0163	157.42±3.49	9563±212
	Poorer	0.8451***±0.0152	0.6205***±0.0195	0.4577***±0.0201	194.98±5.18	11845±315
	Middle	0.7710***±0.0152	0.5335***±0.017	0.3591***±0.0168	224.23±4.72	13622±287
	Richer	0.6947***±0.0182	0.4265***±0.0173	0.2739***±0.0156	264.63±5.21	16076±317
	Richest	0.5322***±0.0211	0.2824***±0.0158	0.1577***±0.0122	386.73***±5.3	23494***±322
**Serial number of thepregnancy**	1	0.7769±0.0070	0.5379±0.0082	0.3671±0.0080	237.63±2.45	14436±149
	2	0.5776±0.3389	0.4606±0.3850	0.2127±0.2172	150.8±50.46	9161±3065
**Type of Health Facility**	Public	0.625±0.0122	0.3499±0.0110	0.1982±0.0089	127.84±1.15	7766±70
	Private	0.9530***±0.0057	0.8041***±0.0113	0.6426***±0.0141	405.65***±1.98	24643***±120
**Region**	North	0.7213±0.0190	0.4835±0.0223	0.2985±0.0214	198.27±6.36	12045±387
	Central	0.6900**±0.0197	0.4392***±0.0214	0.2893±0.0197	184.74±6.09	11223±370
	East	0.8173***±0.0162	0.5481***±0.0201	0.3712***±0.0197	198.17***±5.21	12039***±317
	Northeast	0.8872***±0.0149	0.6858***±0.0227	0.4946***±0.0254	228.69***±5.6	13893***±340
	West	0.7212±0.0229	0.4838±0.0234	0.3246**±0.0218	270.04±6.43	16405±391
	South	0.8074***±0.0161	0.5814***±0.0185	0.4138***±0.0180	287.44***±5.41	17462***±329
	Union Territories	0.7651**±0.0343	0.5346**±0.0372	0.3913***±0.0348	255.47***±9.93	15520***±603

Significance level

*p<0.05

**p<0.01

***p<0.001; ± value of predicted probabilities or means calculated as upper limit-lower limit of 95% confidence interval divided by 2.

Along with education, economic status, type of health facility and place of residence are also making a significant contribution to catastrophic spending. Residing in urban areas increases the probability of catastrophic spending by 10 percentage points. Similarly, the probability of catastrophic spending for residents in the Southern region is higher by ten percentage points as compared to those living in Central region. Finally, the predicted mean of absolute expenditure in Model–II shows that women living in urban areas, education level upto graduation or above, belonging to other religious groups and General *Caste*, richest MPCE quintile, women delivering in private hospitals were spending relatively more.

## Discussion

Despite making substantial progress towards improving the maternal health, many countries in South Asia and Sub-Saharan Africa continue to face high maternal mortality rates and have failed to meet the target of reducing the MMR up to three-quarters by the end of 2015 under Millennium Development Goal-5 (MDG-5). The latest report by the UNICEF, WHO, World Bank and UNDP [1] has argued that an important reason for the failure to achieve MDG-5 target is the lack of access to better quality maternal health care services at affordable prices in these countries. Furthermore, use of maternal health care services across the world and in India is directly or indirectly associated with women’s socioeconomic and demographic status. A number of studies from developing countries have documented the role of socioeconomic disadvantage, particularly at low-income levels as being a major constraint in accessing better quality maternal health care [10–16, 38, 39, 41–46]. In this context, our study has provided a quantitative analysis using the latest data on maternal health care spending in India, and has also documented the critical socio-economic and demographic factors influencing the OOPE and CME of the households. Below, we discuss the critical findings of this study and their relative merits compared to previous studies on the subject.

### Average Cost of Maternity Care

The average cost of delivery (US$ 159 in current price and US$ 92 in constant) and total maternity spending (US$ 258 in current price and US$ 149 in constant price) estimated in this study is significantly higher than the average delivery expenditure (US$ 33) and maternity expenditure (US$ 50.5) estimates of Bonu et al. [4] and delivery expenditure (US$ 44) of Mohanty and Srivastava [25]. The difference can be attributed to two factors: (1) Underestimation of maternity spending in previous national-level studies possibly due to the limitations of the data they have used; (2) The real expenditure on maternity care services increased in 2014 in comparison with the earlier period, 2004–05 because of an increase in the use of private health care and the number of caesarean delivery cases in 2014 compared to 2004–05 [27, 28]. An increase in public awareness on health has also led to a greater willingness to invest in better maternity health care [5, 47–50].

The evidence on the OOPE on delivery and maternity care are also available at the regional and local level. A comparative assessment of delivery spending in the present study with that of spending on delivery care reported in small-scale studies provides mixed results. For instance, relative to the delivery expenditures estimated in our study (US$ 92), previous studies such as Dhar et al. [48] reported significantly higher delivery expenditure (US$ 370.7), while Shukla et al. [51] found evidence of considerably lower delivery expenditures (US$ 24). Furthermore, a comparison of the influence of OOPE on delivery expenses reported in the present study to that of previous studies from other countries suggest that OOPE on delivery is much lower in African countries like Kenya (US$ 18.4), Burkina Faso (US$ 7.9) and Tanzania (US$ 5.1), but is significantly higher in developed countries such as Canada (US$ 2733) [49, 50]. The reason behind the lower per capita delivery cost in Kenya, Burkina Faso, Tanzania compared to India maybe because they provide greater subsidised maternity care services relative to India. For instance, the share of health expenditure in GDP is 4.5%, 6.4% 7.3% in Kenya, Burkina Faso, and Tanzania respectively, while it is only 4% in India [49].

### Catastrophic Spending and Its Factors

Our findings on CME (51%) from the 71^st^ round of the NSSO is much higher than the CME estimates (16%) in Bonu et al. [24] using the 60^th^ round of NSSO data. Again, this difference may be attributed to an underestimation of maternity expenditure in the 60^th^ round. Another study by Shukla et al. [51] reported that spending on institutional delivery for 81% of household in rural Lucknow was catastrophic. The high CME estimates in their study might be peculiar to their sample and may not be generalized because their findings were based on a small sample and they also used a different definition to measure CME. However, empirical evidence from other developing countries is in tune with our findings. Most of these studies suggest that high OOPE for maternity care can cause households to incur catastrophic expenditures, especially in lower socioeconomic groups which in turn can push them into poverty [50].

In the order of their importance, economic and educational status, place of residence, type of health facility, social affiliation of women and her age have emerged as significant predictors of CME. Further, our findings are in tune with previous studies [24, 14, 25, 26]. The new addition from this study is that these factors were identified using comprehensive disaggregated information on maternity care expenditure that includes spending on ANCs, delivery care, and PNCs, and our results are robust.

The availability of information on disaggregated maternity expenditure in the 71^st^ round of NSS did overcome some of the serious limitations that were reported in previous rounds of NSS and DLHS. Despite these strengths, our study has some limitations: (i) the disaggregate information on maternity care expenditure in the 71^st^ round was collected only for delivery care expenditure, while the data on ANCs and PNCs expenditure were collected at an aggregate level. This has the potential to underestimate the absolute level of expenditure compared to estimates based on disaggregated data on each cost item [28]. However, this method is still expected to provide a sensible proxy for relative ranking of households according to the level of their socio-economic standing [24, 27, 28]; (ii) as noted in previous studies, estimated births in the NSS are fewer than the births estimated in other surveys and Census, signifying potential under-reporting of births. The degree of difference in under-reporting of births across diverse population groups may lead to an overestimation or underestimation of average maternity expenditure [24]; (iii) another limitation of this study is the non-availability of information on *JSY* benefits in the recent NSS round. This has prevented us from analysing its contribution in protecting households from CME. Future surveys on maternity health care and related expenditures in India need to consider the limitations highlighted here to adopt suitable steps to overcome them.

## Conclusion

Findings from both the present analysis and previous studies suggest that high OOPE on maternity care can be a serious constraint in utilizing quality maternity care in developing countries. Therefore, some governments in developing countries have initiated demand-side financing schemes to avoid CME burden (e.g. Mexico’s *Oportunidades*; Pakistan and Bangladesh’s *Voucher Scheme*; Peru’s *Juntos*; Bolivia’s *Bono Juana Azurduy*; Nepal’s *Safe Delivery Incentive Program*). Similarly, in India, the *JSY* was launched to reduce maternal and child mortality, and reduce the burden of CME on households. The plan was to increase the use of prenatal care and institutional deliveries in public health facilities to counter the economic burden of CME. Within five years, *JSY* program has made substantial strides with the number of beneficiaries increasing from 0.74 million in 2005–06 to 11 million in 2013–14 [10, 52], thus covering around 40 percent of total deliveries in the country. Its budgetary allocation has also increased from US$ 8.5 million in 2005–06 to US$ 275 million in 2008–09 [10, 53]. Nevertheless, out of 14,482 deliveries analyzed in this study, the results show that the cost of institutional delivery was Zero in only 19 deliveries. Further, it indicates that maternity entitlements for women under the *JSY* scheme is not sufficient to lift households out of catastrophic spending because the mean spending on maternity care (US$ 258) is ten times higher than the *Voucher* amount obtainable under *JSY* entitlements (US$ 23). Even the delivery cost of US$ 160 or Rs. 9701 is significantly higher than the *JSY* entitlement (S1 Table). Moreover, these are only direct expenditures. Apart from this, there are also indirect costs which need to be factored in women and some of their family members who escorted her may also lose their wages during pregnancy and delivery, which may impact negatively on households in the lower socio-economic status.

Thus, India needs to prioritise its rising public health spending in general and *JSY* entitlements, in particular, to address the exceedingly high OOPE that many women incur for maternity care. The public health spending in India (4% of GDP) is among the lowest, not only in the larger economies of the world, but also compared to some of the poorer countries such as Malawi (8.3%), Namibia (7.7%), Tanzania (7.3%) and Sudan (6.5%) [54–57]. Given the large disparities in the cost of maternity care between public and private health facilities, there is also a need to control the sky-rocketing costs of private health care services, and increase the availability and accessibility of quality public health facilities for maternity care in India. Strengthening birth preparedness strategies and identifying complications during ANCs may reduce the incidence of catastrophic expenses at the time of delivery.

## Supporting Information

S1 Table Mean spending (Rs.) on maternity care services. (DOCX)
