# Supplementary material for: High Spending on Maternity Care in India: What Are the Factors Explaining It?
Source: PLoS One. 2016 Jun 24;11(6):e0156437. doi: 10.1371/journal.pone.0156437 (PMC4920397; doi:10.1371/journal.pone.0156437)
Supplement: S1 Table — (DOCX) [file pone.0156437.s001.docx]

**S1 Table. Mean spending (Rs.) on maternity care services.**

| Variable | Categories | Prenatal | | Postnatal care | | Delivery cost | | Total maternity cost | |
| --- | --- | --- | --- | --- | --- | --- | --- | --- | --- |
|  |  | Mean | ±CI | Mean | ±CI | Mean | ±CI | Mean | ±CI |
|  |  | (n= 13596) |  | (n=10798) |  | (n=14482) |  | (n=10379) |  |
| Age (in years) | 15-24 | 2655 | ±246 | 1634 | ±125 | 9839 | ±836 | 14302 | ±1021 |
|  | 25-29 | 2895 | ±193 | 1815 | ±121 | 10002 | ±1174 | 14677 | ±703 |
|  | 30-49 | 2918 | ±335 | 1718 | ±151 | 8979 | ±736 | 13663 | ±878 |
| Place of residence | Rural | 2268 | ±181 | 1552 | ±93 | 8418 | ±719 | 11924 | ±679 |
|  | Urban | 4097 | ±232 | 2106 | ±132 | 13068 | ±795 | 19801 | ±742 |
| Education Level of women | No education | 2307 | ±231 | 1692 | ±182 | 6152 | ±627 | 10191 | ±706 |
|  | Primary | 2880 | ±353 | 1657 | ±127 | 8447 | ±811 | 12888 | ±879 |
|  | Secondary | 2599 | ±295 | 1634 | ±182 | 8994 | ±1838 | 12401 | ±890 |
|  | Higher secondary | 3049 | ±334 | 1724 | ±139 | 11033 | ±1254 | 15481 | ±811 |
|  | Graduate and above | 3505 | ±442 | 1978 | ±258 | 19577 | ±2409 | 27050 | ±3336 |
| Religion | Hindu | 2713 | ±163 | 1684 | ±88 | 9711 | ±612 | 14269 | ±651 |
|  | Muslim | 3047 | ±452 | 1758 | ±179 | 9692 | ±2125 | 13358 | ±900 |
|  | Others | 3228 | ±458 | 1960 | ±240 | 9612 | ±907 | 16117 | ±1353 |
| Social group | SC/ST | 2486 | ±213 | 1821 | ±169 | 6598 | ±1189 | 10679 | ±1258 |
|  | OBC | 2773 | ±217 | 1658 | ±110 | 10821 | ±897 | 14920 | ±713 |
|  | General caste | 3143 | ±334 | 1685 | ±118 | 11511 | ±841 | 17178 | ±868 |
| MPCE quintile | Poorest | 2508 | ±250 | 1687 | ±188 | 4793 | ±441 | 9321 | ±616 |
|  | Poorer | 2629 | ±379 | 1635 | ±172 | 8361 | ±1950 | 12771 | ±2242 |
|  | Middle | 2899 | ±387 | 1683 | ±151 | 8710 | ±711 | 13116 | ±835 |
|  | Richer | 2701 | ±327 | 1751 | ±169 | 11418 | ±1064 | 15892 | ±959 |
|  | Richest | 3343 | ±292 | 1828 | ±153 | 17656 | ±1829 | 22191 | ±1166 |
| Serial number of the pregnancy | One | 2792 | ±148 | 1712 | ±76 | 9700 | ±565 | 14288 | ±543 |
|  | Two or more | 1710 | ±915 | 5821 | ±7301 | 10447 | ±7343 | 6790 | ±2188 |
| Place of delivery | Public | 2128 | ±183 | 1216 | ±73 | 8728 | ±426 | 11986 | ±494 |
|  | Private | 5160 | ±354 | 2678 | ±178 | 10586 | ±615 | 18588 | ±806 |
| Insurance | No | 2777 | ±357 | 1655 | ±142 | 8695 | ±870 | 13336 | ±765 |
|  | Yes | 3446 | ±684 | 2225 | ±623 | 19670 | ±3702 | 26720 | ±3877 |
| Region | North | 2666 | ±277 | 1373 | ±166 | 7102 | ±686 | 12097 | ±861 |
|  | Central | 2988 | ±466 | 1633 | ±197 | 6612 | ±989 | 10596 | ±986 |
|  | East | 2975 | ±439 | 1747 | ±257 | 7586 | ±1613 | 13178 | ±2541 |
|  | Northeast | 3485 | ±453 | 2362 | ±330 | 7303 | ±691 | 14252 | ±1112 |
|  | West | 2543 | ±370 | 1890 | ±200 | 11530 | ±1387 | 14971 | ±1158 |
|  | South | 2808 | ±306 | 1688 | ±132 | 13080 | ±1490 | 17309 | ±989 |
|  | Union Territories | 2126 | ±432 | 1711 | ±310 | 11580 | ±1579 | 15259 | ±2018 |
| Total |  | 2791 | ±148 | 1714 | ±77 | 9701 | ±565 | 14286 | ±543 |
